# Bake-Out Strategy Considering Energy Consumption for Improvement of Indoor Air Quality in Floor Heating Environments

**DOI:** 10.3390/ijerph15122720

**Published:** 2018-12-03

**Authors:** Seonghyun Park, Janghoo Seo

**Affiliations:** 1 Department of Architecture, Graduated school, Kookmin University, 77 Jeongneung-ro, Seongbuk-gu, Seoul 02707, Korea; marine86@kookmin.ac.kr; 2 School of Architure, Kookmin University, 77 Jeongneung-ro, Seongbuk-gu, Seoul 02707, Korea

**Keywords:** bake-out, indoor air quality, computational fluid dynamics, toluene, floor heating system

## Abstract

Improved quality of life has led to a growing demand for better indoor air quality (IAQ). Buildings are becoming more airtight and insulated in order to minimize energy consumption. The importance of both energy conservation and IAQ improvement has been recognized and addressed by many studies. Bake-out is the process of using indoor heating to remove volatile compounds present in building materials and furnishings so that they can be vented out into the atmosphere. Indiscriminate use of heating to increase the surface temperature of materials during this process can result in significant loss of energy. Therefore, energy-efficient bake-out should be performed by considering both the floor temperature and the emission amount of pollutants. This study aims to investigate an effective and economical bake-out implementation strategy via experimentation and computational fluid dynamics analysis. The results showed weak direct correlation between the heating energy consumption and the amount of pollutants emitted. The study also highlights the passive option of installing sorptive building materials for improving IAQ economically.

## 1. Introduction

Apartment houses and other newly constructed buildings are ensuring increased airtightness and insulation in their structures by using building materials made of complex chemicals in order to reduce energy consumption. However, volatile organic compounds (VOCs) such as formaldehyde (HCHO) and toluene emitted from building materials composed of complex chemicals degrade the indoor air quality. Workers or occupants continuously exposed to these environments, can contract lung cancer or respiratory diseases [1,2,3,4]. Therefore, it is necessary to make efforts to improve indoor air quality (IAQ). The methods for IAQ enhancement can be categorized into elimination, source removal and dilution. Bake-out, which is one of the elimination methods, has drawn considerable attention and it is widely used to reduce indoor air pollutant concentration before occupants move into newly built residences. Much research has been conducted on bake-out and is still ongoing [5,6]. Lu et al. [7] conducted an empirical study on VOC elimination technology employing the chamber method for households in China and noted that bake-out performance is greatly influenced by the temperature and ventilation period. In addition, their study presents an ideal ventilation frequency for the optimal elimination of VOCs. Edwards et al. [8] studied the health effects of indoor air condition on occupants by taking samples of 30 VOCs in the air and analyzing the similarities between their pollutants. Kang et al. [9] studied the bake-out effect of floor heating systems in South Korean households. Small-scale chamber experiments were conducted to gauge the amount of VOC emissions and a quantitative result was derived showing that shorter bake-out periods correspond to lesser effectiveness in reducing emissions. Lv et al. [10] investigated the effects of bake-out with dilution ventilation technology using experimental and numerical methods. These effects are influenced by bake-out temperatures, times and ventilated times. Lee et al. [11] presented an analytical model for the sinking behaviors of VOCs against porous building materials. Jiang et al. [12] investigated the emission characteristics of HCHO and VOCs using a particle board with high VOC emission in sealed and ventilated environmental chambers.

The existing studies place much emphasis on identifying the emission characteristics of VOCs for different materials through surveys and experiments and implementing bake-out to remove these pollutants for better IAQ. It can be inferred from most of these findings that the elimination of VOCs from building materials can be accelerated by increasing the indoor temperature and the amount of ventilation using mechanical devices. However, these studies tend to focus only on enhancing the bake-out performance for reducing pollutant concentration. Although these methods may be effective for IAQ improvement, they could result in financial losses if too much energy is used in the process. Sorptive building materials (SBM), which are considered a passive method for pollutant adsorption, are an efficient tool that can reduce VOC concentration while replacing the existing building material at the same time. These materials remove chemical substances from the indoor air by physical sorption or chemical reaction [13]. Seo et al. [14] evaluated the effects of SBM on the reduction of VOCs through an experiment using a small chamber. Park et al. [15] explored effective installation methods of SBM in office environments by measuring and analyzing their VOC reduction effect per installation area based on occupant satisfaction. These methods reflect the need to develop an efficient and economical bake-out strategy for IAQ improvement by comparatively analyzing the cost of energy used during bake-out periods and reduction in VOC concentration.

In this study, test chamber experiments and computational fluid dynamics (CFD) analysis are conducted by breaking down the bake-out procedure into two steps and measuring the changes in VOC concentration under different variables for each step. This study also analyzes the pollutant reduction efficiency in terms of the amount of energy used and reexamines the economic feasibility and practicality of the existing bake-out strategy, which focuses on performance.

## 2. Materials and Methods

This study divides the bake-out procedure into two parts and conducts experimentation and CFD analysis. In the first step, the VOC concentration is measured using different variables in sealed mock-up chambers with an insulated floor, simulating the “bake” portion of the process. In the second step, the effectiveness of natural ventilation, mechanical ventilation and sorptive building materials are analyzed using CFD analysis, representing the “bake-out” portion.

Table 1 shows the heating and bake-out process conditions for each case. The floor temperature was set to 30, 40 and 50 °C and the VOC concentration was analyzed for each instance. For all cases, 40 mL of pollutant was injected.

To evaluate the performance of pollutant concentration reduction by bake-out, three alternatives (ALTs) were set. The bake-out process is divided into natural ventilation, mechanical ventilation and installation of SBM. 

### 2.1. Experimental Conditions

#### 2.1.1. Target Space and Experiment Method

**Figure 1:**
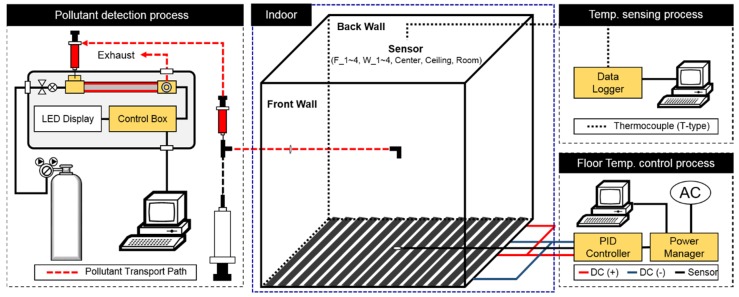
Diagram of the Experiment.

Figure 1 illustrates an overview of the experiment. The experimental apparatus consists of the test chamber, a pollutant detection device, a floor temperature control device and a temperature sensor. The test chamber is a cube measuring 1800 mm on each side. It was built using Isopink for thermal insulation purposes and the edges were sealed airtight with silicon. The VOC detector was filled manually, which means that a sampling device was required to measure the level of VOC concentration at the center of the chamber. Stainless Steel (SUS 316, KWANGSHIN I.S.T Inc., Hamyang, Korea), which has no VOC emission, was used for the construction of the sample injection tube and the piping union and a 100 mL medical syringe was used as an air pump. The measurement interval was set to 10 min.

**Figure 2:**
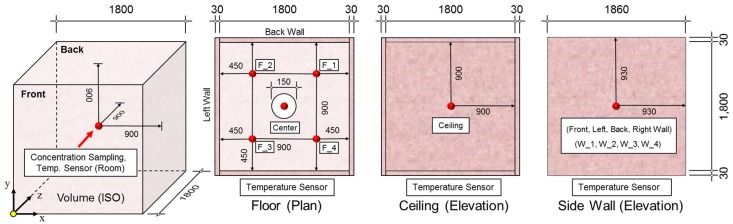
Sensor locations within the chamber.

Figure 2 shows the temperature sensor locations within the chamber. A total of 11 sensors were used to ensure uniform temperature control: four on the floor (F_1–4) and one at the center of each side wall as well as the ceiling. In addition, there was a concentration sampler at the center of the chamber.

Table 2 shows the details of the experimental equipment used for the experiment in this study. A positive temperature coefficient (PTC) film was used to simulate floor heating in order to produce an even control of temperature and the heating was supplied electrically. This made it possible to calculate the amount of energy used for temperature control and maintenance for each case.

To simulate the presence of pollutants, a total of 40 g of adhesives (two Loctite 401), which is highly concentrated in VOC, was sprayed on the floor and allowed to dissipate.

Table 3 lists the specifications of the VOC analyzer (SGVA-P2, FIS Inc., Itami, Japan) used in this study. This VOC analyzer is a gas-chromatography-type gas-concentration-measuring device that uses a metal–oxide–semiconductor gas sensor and employs high-purity air as the carrier gas. The compounds separated though the columns are detected using a semiconductor gas sensor. Finally, the concentrations of the target gas are automatically calculated and indicated. The correlation of the results obtained from this analyzer with those obtained from the gas chromatography/mass spectrometry (GC/MS) method was more than 0.99 [16,17,18]. Samples for the VOC analysis were obtained every 10 min, considering the performance of the VOC analyzer (8 min measurement time). As there was a difference in the distance between the center of the chamber and the sampling point, the volume of air corresponding to the internal volume of the sampling tube was collected for the first time using a sterile syringe. Thereafter, a sampling syringe was used to obtain a 5 cc sample for the second time and to insert it into the VOC analyzer.

The experiment lasted for 10 h and included an indoor cooling period, temperature stabilization and pollutant measurement. To fully eliminate residual pollutants within the chamber after the experiment of each case, experiments were conducted at intervals of one week per case (total of three weeks) and the background concentration before measurement was measured. 

#### 2.1.2. Floor Temperature Control

The energy used for heating must be quantitatively derived in order to analyze the effectiveness of baking at varying degrees of energy consumption. This study employed a PTC heating film to obtain an even distribution of heat across the floor. The floor temperature was controlled using the PID control method, which can deliver timely response to the input values and has a small offset through the use of proportional, integral and differential parameters. Its basic formula is given in Equation (1). In addition, the input variables of proportional (P), integral (I) and differential (D) for the PID controller were set to 5%, 60 s and 15 s, respectively.

(1) $${\;\beta (t)\;=\;K}_{p}\left(e\left(t\right)+\frac{1}{T_{i}}\int e\left(t\right)dt+T_{d}\frac{de\left(t\right)}{dt}\right)$$

where *β* is the output value, *e* is the error factor, *K_p_* is the proportional gain, *T_i_* is the integral time and *T_d_* is the differential time.

A T-type thermocouple was used to measure the temperature while the Data Logger (GL 220, GRAPHTEC Co., Yokohama, Japan) kept track of the data and monitored the values. The instantaneous and total energy consumption of the system were measured using a Power Manager (B200S, DAWONDNS Co., Gwangju, Korea). The room outside the chamber was cooled to a set temperature of 25 °C for 30 min in order to keep the temperature outside the chamber static. Then, a heating source was used to measure the temperature change and energy consumption. The changes in temperature were measured in intervals of 10 s.

### 2.2. Numerical Model of CFD Analysis

#### 2.2.1. Boundary Condition of CFD Analysis

This study aims to analyze the pollutant concentration using CFD analysis and develop an efficient bake-out strategy. The concentration of VOC emissions from building materials or adhesives is influenced internally by the physical and chemical properties of the material and externally by temperature, air velocity, turbulence intensity and relative humidity. This calls for a sufficient amount of information regarding these influencing factors. However, the existing method for measuring VOC concentration relies on a single variable and only assesses how an individual factor affects the ventilation characteristics [19]. Numerical analysis of CFD allows the assessment of pollutant emission in the air under different variables using various models. 

**Figure 3:**
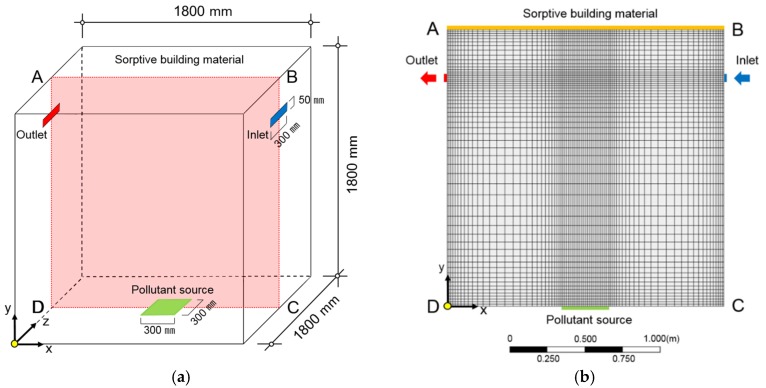
Model used for computational fluid dynamics (CFD) analysis: (**a**) Geometry; (**b**) Mesh (Section ABCD).

Figure 3 shows the target space and mesh for the CFD analysis. This space was assumed to have the same size as the experimental space, with an inlet and an outlet set on the sides for ventilation. In addition, the SBM was assumed to be installed on the entire surface of the ceiling.

Table 4 shows the boundary conditions for the CFD analysis used in this study. ANSYS Workbench 17.2 and FLUENT CFD engines were used for the analysis [20]. There were approximately 400,000 mesh elements and a no-slip condition finish was applied to the sidewalls of the test chamber. The dimensions of the space, the temperature of the test chamber walls, the heated temperature of the floor and the pollutant set values were set according to the experiment. All interpretations were performed in an unsteady state to observe the changes in pollutant concentration over time.

In the case of natural ventilation and mechanical ventilation, the inlet boundary conditions of the airflow were calculated to correspond to air changes per hour (ACH) of 0.5 h^−1^ and 3 h^−1^, respectively. A separate velocity profile was written for mechanical ventilation and the maximum wind velocity was set to 0.53 m/s. Natural ventilation and SBM installation methods are passive methods and do not consume energy. Furthermore, the SBM installation method has excellent concentration reduction performance against volatile pollutants, such as toluene, without incurring any additional operating cost. Toluene, which was found in the highest concentration during the experiment, was used as the pollutant. The analysis was conducted for 2 h in the unsteady state.

#### 2.2.2. Turbulence Model and Governing Equations for CFD Analysis

According to details from existing studies, the SST k-ω model can predict the turbulence accurately under the advanced wall function condition when the Y + value is less than 10 [21]. On the other hand, when the Y + value is less than 300, the standard k-e model can be applied to improve the convenience of input parameters and lattice configuration. However, in this case, the inaccuracies in flow prediction at the separation, swirling flow and nearest wall should be considered. Thus, we used a low Reynolds number k-ε model instead of the standard k-ε model, which is known to accurately predict the natural convection due to buoyancy and small changes in the near-wall turbulent energy [22]. The fluid in the space was set as an incompressible ideal gas and the buoyancy was set to be generated according to the density change. The continuity, momentum and energy equations are shown in Equations (2)–(4).

(2) $$\frac{\partial \rho }{\partial t}+\nabla \cdot \left(\rho u\right)\;=\;0$$

(3) $$\frac{\partial \left(\rho u\right)}{\partial t}+\nabla \cdot \left(\rho uu\right)\;=\;-\nabla p+\rho g+\nabla \cdot (\mu \nabla u)-\nabla \cdot \tau_{t}$$

(4) $$\frac{\partial \left(\rho e\right)}{\partial t}+\nabla \cdot \left(\rho eu\right)\;=\;\nabla \cdot {(k}_{eff}\nabla T)-\nabla \cdot (\underset{i}{\sum }h_{i}j_{i})$$

where *ρ* is the density of the fluid, *t* is the time, *u* is the fluid velocity vector, *p* is the pressure, *g* is the vector of gravitational acceleration, *μ* is the molecular dynamic viscosity, *e* is the specific internal energy, *k_eff_* is the effective heat conductivity, *T* is the fluid temperature, *h_i_* is the specific enthalpy of the fluid and *j_i_* is the mass flux of the *i*-th constituent. The last term on the right hand side of Equation (3) is the divergence of the turbulence stresses (Reynolds stresses) and *τ_t_*, accounts for the auxiliary stresses due to velocity fluctuations.

#### 2.2.3. Pollutant Diffusion Model

This study employed a species transport model for the dissemination of pollutants. The atmosphere inside the test space comprised air gas, simulating fresh outdoor air and toluene. Each constituent gas was set as an incompressible ideal gas with a temperature dependency. IDs were given to the emitted pollutants to enable quantitative measurement of the changes in their concentration. The partial differential transport equation, which was used to measure the pollutant concentration when the ID-ed pollutant passed through the Control Volume (CV) of the three-dimensional space, was derived from Equation (5) [23].

(5) $$\frac{\partial Y_{i}}{\partial t}+\nabla \cdot \left({\rho Y}_{i}u\right)\;=\;-\nabla \cdot j_{i}$$

where *Y_i_* is the mass fraction of the *i*-th air constituent. Due to the reasonably low thermal parameters (pressure and temperature) of air, it can be treated as a rarified mixture. Hence, the mass flux of the *i*-th constituent may be calculated using Equation (6).

(6) $$j_{i}\;=\;-D_{eff}\nabla \cdot Y_{i}$$

where *D_eff_* is the effective diffusion coefficient, which includes the turbulence effects.

The pollutant concentrations were calculated under the premise that the target pollutants are passive. Passive pollutant analysis is a simple method for calculating the pollutant distribution. It assumes that the pollutant and air share the same properties and generally defines the Schmidt number (=*v/D_a_*) as 1.0. However, the diffusion coefficient *D_a_* should be considered because it is an important boundary condition that affects the diffusion of the pollutant concentration in CFD analysis. In general, Hirschfelder equation is widely used to calculate the air diffusion coefficient; however, it is often inconsistent with the experimental value. In this study, the Fujita equation is used to derive and apply the diffusion coefficient of toluene under different temperatures. 

The diffusion of toluene and the analysis of sorption were performed and toluene was assumed to be a passive contaminant. The concentration distribution was produced and the water (vapor) diffusion in air was expressed as shown in Equation (7). The water (vapor) diffusion coefficient in air was calculated using Equations (8)–(10) [24,25].

(7) $$\frac{\partial \bar{C_{1}}}{\partial t}+\frac{\partial \bar{U_{i}C_{1}}}{\partial x_{i}}\;=\;\frac{\partial }{\partial x_{i}}\left(\left(D_{a}+\frac{v_{t}}{{Sc}_{t}}\right)\frac{\partial \bar{C_{1}}}{\partial x_{i}}\right)$$

(8) $${log}_{10}P_{w}\;=\;\frac{A-B}{C+T}-3$$

(9) $$C_{0}{\;=\;\rho }_{a}\times \frac{M_{1}}{M_{2}}\times \frac{P_{w}}{P-P_{w}}$$

(10) $$D_{a}\;=\;\frac{{6.7\times 10}^{-8}{\times T}^{1.83}}{P}\times {\left[{\left(\frac{T_{c1}}{P_{c1}}\right)}^{1/3}+{\left(\frac{T_{c2}}{P_{c2}}\right)}^{1/3}\right]}^{-3}\sqrt{\frac{1}{M_{1}}+\frac{1}{M_{2}}}$$

where *C*_1_ is the pollutant concentration at a spatial point (μg/m^3^), *D_a_* is the molecular pollutant diffusion coefficient (m^2^/s), *U_i_* is the wind velocity (m/s), *v_t_* is the eddy viscosity (m^2^/s), *Sc_t_* is the turbulent Schmidt’s number (-) and *P_w_* is the water vapor pressure (Pa). In addition, A, B and C are the empirical constants with values of 7.7423, 1554.16 and 219, respectively. T denotes the temperature (°C), *C*_0_ is the saturation concentration (g/m^3^) and *ρ_a_* is the air density (g/m^3^). *M*_1_ and *M*_2_ are the molecular weights, P is the atmospheric pressure in the chamber (Pa), *T_c_*_1_ and *T_c_*_2_ are the critical temperatures (°C) and *P_c_*_1_ and *P_c_*_2_ are the critical pressure values (Pa). 

#### 2.2.4. Pollutant Adsorption Model

This study used CFD analysis to assess the reduction in contaminant concentration when SBM is used in the bake-out process. The pollutant adsorption model was examined and applied to the CFD analysis. The adsorption of contaminants was measured under the premise that the surface density *C_s_* of SBM is zero. This is consistent with the Henry constant *K_h_* = ∞ in the Henry adsorption isotherm, which is used when the adsorbent in questions has an outstanding adsorption effect. In this study, the adsorbent flux adsorption model for the surface of SBM was applied to a single material. The adsorption model was applied to homogeneous materials and the adsorption flux, or *flux_ad_*, on SMB surfaces was found using Equation (11) [26].

(11) $${flux}_{ad}=-D_{eff}{\frac{\partial C}{\partial x}|}_{B}=-D_{eff}\frac{C_{CV}-C_{eq}}{∆x}$$

where *C_CV_* is the contaminant concentration of the first control volume (CV) of air coming in contact with the SBM surface (μg/m^3^), *C_eq_* is the SBM surface′s adsorption concentration (μg/m^3^) and *D_eff_* is the effective diffusion coefficient (m^2^/s).

## 3. Results and Discussion

### 3.1. Floor Temperature Control and Energy Consumption

**Figure 4:**
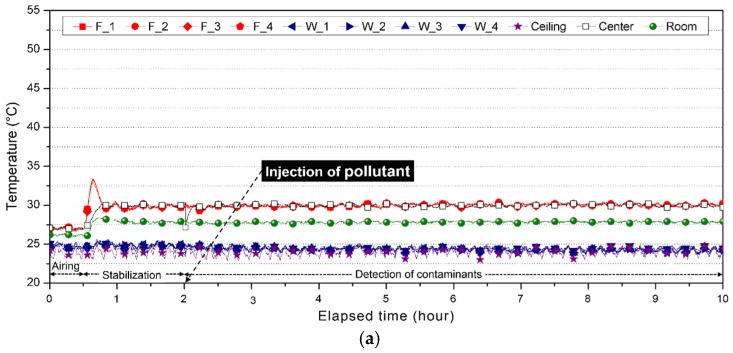

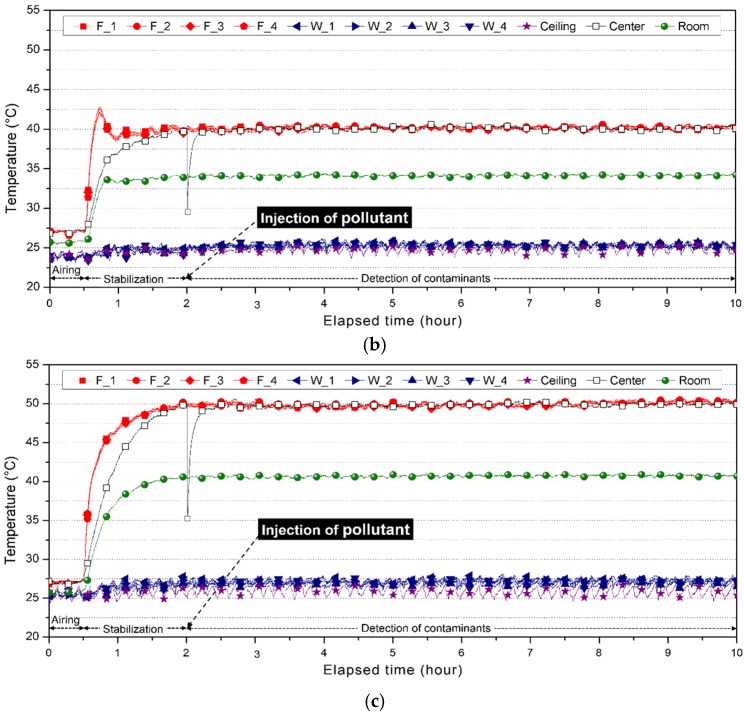
Temperature change over time per case: (**a**) Case 1 (Floor heating set temp. 30 °C); (**b**) Case 2 (Floor heating set temp. 40 °C); (**c**) Case 3 (Floor heating set temp. 50 °C).

Figure 4 shows the temperature change in the sensors for each case over time. Heating was turned off during the first 30 min of the experiment and Cases 1–3 showed similar temperature changes when cooling by airing was administered under identical conditions. As the results show, a dramatic change in temperature was detected once heating was turned on and the floor temperatures reached steady states in relation to the set values within 30 min in all cases. This is typical of PID control that has a high initial response speed.

In Case 1, where the floor temperature was set to 30 °C, both the maximum and minimum temperatures of all sensors were within ±0.8 °C from the set values with only small differences. The average temperature was measured to be identical to the set value at 30 °C and the standard deviation and range were found to be similar across all sensors. This situation occurs owing to the target value′s high proximity to the set value as a result of PID control as well as the presence of very little offset. On the other hand, the temperature distribution of Case 2 shows a slight increase in the range of oscillation with respect to the measured temperature based on the set value. However, considering that the peak load of a typical on/off control is ±4 °C, floor heating by PID control and PTC film was deemed reliable for maintaining the set temperature.

**Figure 5:**
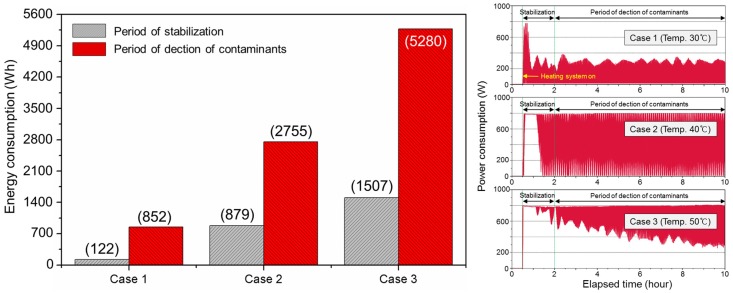
Energy and power consumption from heating under PID (proportional integral derivative) control each Case.

Figure 5 shows the energy and power consumption over time per case. Maximum instantaneous energy consumption was observed immediately after heating was turned on and its oscillation range subsequently lowered as the target value approached the set value. In Case 1, the initial manipulated variable did not show dramatic change in relation to the set time for the derivative control of PID. For Case 3, on the other hand, a substantial change in the manipulated variable was seen during the set time and maximum energy was continuously supplied for about 1 h after the heating was turned on. The energy consumption for Case 1 with floor surface temperature set at 30 °C was 974 Wh. On the other hand, the total energy consumption in Case 2, whose difference in floor temperature was 13 °C before and after the heating, was 3634 Wh, which is about 2.7 times greater than that of Case 1.

### 3.2. Quality Assurance of Pollutant Measurement and Analysis

This study validated pollutant measurement and analysis by assessing linearity, precision and accuracy per the ICH Guidelines [27].

Linearity is related to the accuracy and scope of the analysis and in this study plots of signal strength against concentrations of VOCs were visually evaluated. When a linear relationship was recognized, the measurement method was assessed using statistical methods, such as calculating the slope of the line using the least squares method. A total of six VOC concentrations were used for the study: 5 ppb, 10 ppb, 30 ppb, 100 ppb, 300 ppb and 1000 ppb.

**Figure 6:**
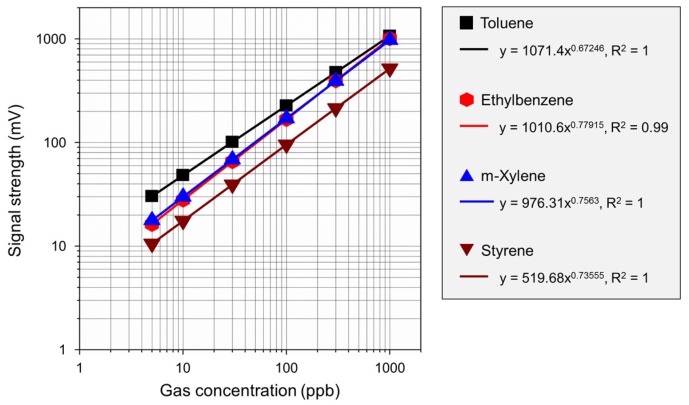
Calibration curve of VOCs (Toluene, Ethylbenzene, m-Xylene and Styrene).

Figure 6 shows the linear calibration curves of each VOC fit according to the least squares method. The correlation coefficient (R^2^) was greater than 0.99 for all of them. Hear, the relation should be linear in the log-log scale because of semiconductor characteristics.

To verify precision, retention time and signal strength were compared after measuring VOCs with identical gas concentrations eight times. Table 5 shows the mean signal strength and concentration of toluene, ethylbenzene, m-xylene and styrene. The relative standard deviations (RSD) of the VOCs were moderate, ranging from 2.07 to 2.82%.

Accuracy was measured by repeatedly measuring four different concentrations four times each (Table 6). The accuracy of the VOCs concentration measurement was between 98.00% and 112.11%, with the relative standard deviation (RSD) < 2.84%. Also, the RSD of measured concentration ranging from 10 ppb to 100 ppb was lower than 1.02%.

### 3.3. Change in Pollutant Concentration According to Floor Temperature (Bake Process)

Table 7 shows the results of 10 measurements of background concentration in the test chamber prior to the injection of pollutant. In Case 3, where the floor temperature was set to 50 °C, more contaminants were emitted compared to the other cases. Thus, it can be deduced that residual contaminants were discharged as the test body was heated. Five pollutant types were detected during the measurement period, in which o-xylene was not included. Further, toluene concentration appeared to be the highest in all cases.

**Figure 7:**
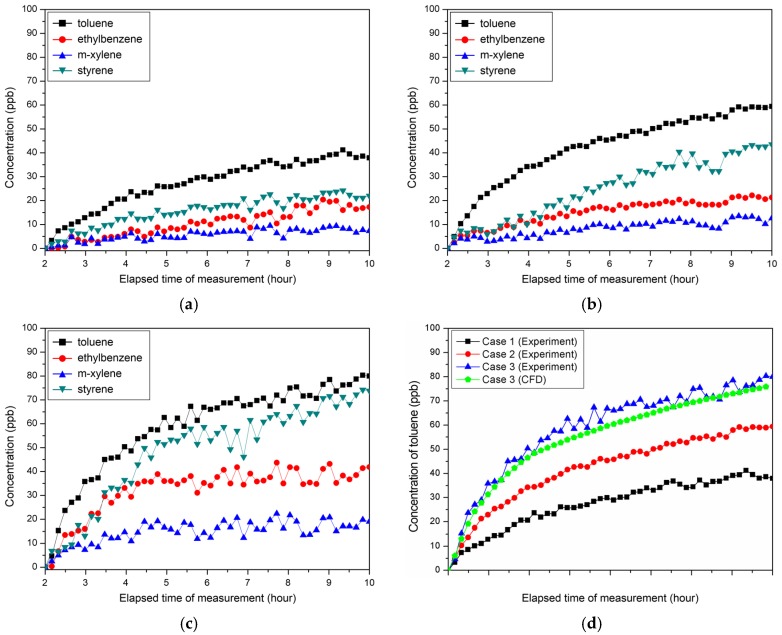
Concentrations of pollutants per case: (**a**) Case 1; (**b**) Case 2; (**c**) Case 3; (**d**) Toluene concentration of each case. CFD: computational fluid dynamics.

Figure 7a–c show the changes in contaminant concentrations per case. Figure 7d illustrates the changes in concentration of toluene and the results of the CFD analysis. It was found that higher floor temperatures lead to higher emission rates across all pollutant types but m-xylene to a lesser degree than others. Thus, measures other than increasing the surface temperature are necessary to reduce the pollutant concentration of m-xylene, which is emitted from building materials. The maximum concentrations of toluene detected in the center of the test chamber during the pollutant measurement period were 41.0 ppb, 59.4 ppb and 80.3 ppb for Case 1, Case 2 and Case 3, respectively. Experimentation and CFD analysis of Case 3 showed that the differences in toluene concentrations were within a 5% margin of error, lending credence to CFD analysis based on the pollution diffusion model employed in this study. 

**Figure 8:**
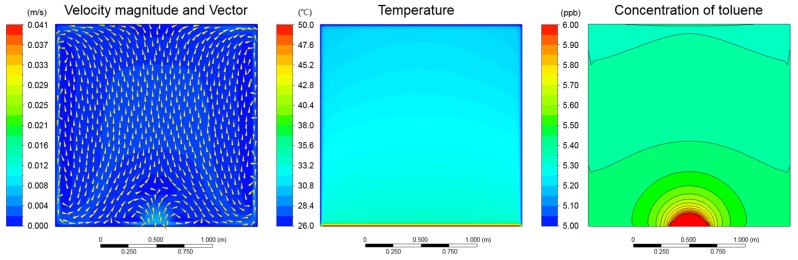
Results of CFD (computational fluid dynamics) analysis (Case 3, time = 600 s, Section XY, Z = 0.9 m).

The CFD analysis of Case 3 at time (600 s) is illustrated in Figure 8. The ΔT value between the floor and side walls were observed to be 25 °C, which resulted in natural convection within the test chamber. The air current ascended at the center of the floor, then cooled down and descended against the side walls. The boundary layer of toluene concentration also disseminated at the center of the floor. However, considering that the average wind velocity in the chamber is 0.02 m/s, it can be concluded that rise in temperature has more effect on toluene diffusion than the natural ventilation.

Table 8 shows the relationship between energy consumption and rate of pollutant concentration increase per case. When the floor heating temperature was set to 50 °C, the amount of energy consumed increased to about five times as that of Case 1 but the maximum concentration of toluene detected increased by a factor of approximately 0.9. This shows that although raising the temperature during the bake-out process helps to increase pollutant emission, the concentrations are not proportionally increased. Hence, a more efficient and economical operation method is needed.

### 3.4. Evaluation of Contaminant Concentration Reduction through Use of Sorptive Building Material and Ventilation (Bake-out Process)

**Figure 9:**
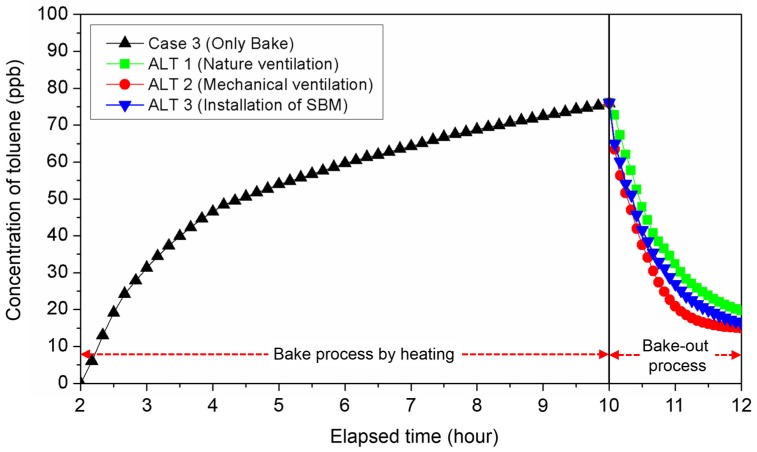
Concentration of pollutant during Bake-out process using CFD (computational fluid dynamics) analysis. SBM: sorptive building material.

Figure 9 shows the changes in toluene concentration per ALT during bake-out. The toluene concentration in the chamber continuously increased for 8 h during floor heating and immediately after the bake-out started, the toluene concentration decreased rapidly. As a result, very low toluene concentrations were observed after 2 h regardless of the bake-out method.

Table 9 shows the results of the step-down tracer gas test to compare the energy consumption (*E*_m_ with fan energy), age-of-air and energy consumption of each ALT that occurred during the local age-of-air and bake-out processes. Local age-of-air is a measure proposed by ASHRAE Standard 129 to evaluate the ventilation efficiency of a target space and can be calculated using Equation (12) [28].

(12) $$A_{i}\;=\;\nabla \tau \cdot C_{i,avg}/C_{i,start}$$

where ∇τ is time period of tracer gas measurement, *C_i,avg_* is the average tracer gas concentration at location *i* during ∇τ and *C_i,start_* is the initial tracer gas concentration at location *i.*

Equation (12) enables calculation of the local age-of-air by defining the entire space as a single CV in the CFD analysis. Even if the target space is airtight, reduction in the concentration of indoor air pollutant due to SBM installation and air filtering can be interpreted as synonymous of a ventilation effect. In such a case, a low age-of-air value implies high effectiveness in reducing contamination, similar to ventilation.

In contrast, this study calculated the age-of-air by allocating the center of the test chamber, which is in the same position as in the experiment, as local. The age-of-air was 1605 s when bake-out was performed via mechanical ventilation. Further, it was 1936 s with installation of SBM (ALT 3) and 2293 s with natural ventilation (ALT 1). Although ventilation was not performed with the installation of SBM, the SBM had a pollutant concentration reduction effect similar to ventilation as it adsorbed pollutants. This means that in an environment such as a closed space where natural ventilation is very poor or non-existent, the indoor pollutant concentration can be reduced by installing an SBM. 

## 4. Conclusions

This study tested the effectiveness of bake-out at reducing indoor VOC concentrations via a two-step process, using experimentation and CFD analysis. Floor heating was implemented using PID control so that pollutant reduction could be measured in relation to the amount of energy consumed. PID control is shown to be effective at controlling the temperature within a narrow range with a very small standard deviation, which provides a reliable boundary condition for steady state analysis and allows for experimental and CFD analyses to be performed simultaneously. 

A test chamber was created to analyze the change in contaminant concentration with floor temperature. Contaminant concentrations increased with the increase in floor temperatures but the induced emission rates were below expectation. These results were very similar to the results of previous studies. In this study, we also examined the adequacy of energy consumption in addition to the improvement of indoor air quality. In South Korea, where most households use floor heating systems, it would be more efficient to maintain a moderate temperature for an extended period of time rather than varying the temperatures.

The present study observed how the bake-out process affected pollutant emissions in airtight spaces and used CFD analysis to analyze the performances of three ALTs. The pollutant diffusion models for CFD analysis were found to correspond closely to the experimental data. The mechanical ventilation system was the most effective at reducing pollutant concentration. Installing SBM was also found to be effective, which implies that in places where natural ventilation is unavailable or the ventilation amount is insignificant, SBM can be used for IAQ improvement. Furthermore, SBM does not require additional cost of operation and prevents heat loss due to inflow of outside air during winter and summer. Consequently, as consuming enormous heating energy to reduce pollutant concentration can cause economic losses, economy and sustainability should be considered in every process of the bake-out. To reduce the pollution produced by household appliances and air-conditioning systems, new generation of low-consumption thermo-acoustic refrigerators, risk of polluting emissions and noise can be used [29]. However, the SBM used in this study was assumed to have infinite capacity and its performance may have been enhanced because the applied area was wider than the spatial volume. Moreover, it must be noted that ventilation is performed not to only remove pollutants; therefore, other factors that may require ventilation should be considered. Subsequent research will employ more variables, including ventilation methods, air changes, vent locations, location of SBM and its surface area and adsorbic capacity of SBM, to find the most efficient and economical method of pollutant reduction. In addition, this study focused on CFD analysis of the bake-out process. Therefore, we will carry out a further study based on experiments to verify the reliability of adsorption, removal and ventilation effects, including the diffusion of pollutants.

## Figures and Tables

**Table 1:** Floor temperature and bake-out conditions per case for bake-out experiment.

Case	Set Floor Temp.	Bake Process (Method)	Bake-out Process (Method)	
Case 1	30 °C	Floor heating (Experimental)	N/A	
Case 2	40 °C			
Case 3	50 °C	Floor heating (Experimental and CFD analysis)	ALT 1	Natural ventilation (CFD analysis)
			ALT 2	Mechanical ventilation (CFD analysis)
			ALT 3	Installation of SBM (CFD analysis)

CFD: computational fluid dynamics; ALT: alternatives; SBM: sorptive building materials; N/A: not applicable.

**Table 2:** Details of the experimental apparatus.

Component	Parameter	Description
Room Condition	Cooling temp.	25 °C
Test chamber	Ceiling height	1800 mm
	Floor area	1800 mm × 1800 mm
	Material	Isopink (30 T)
Experimental Equipment	Temp. sensor	Thermocouple (T-type)
	Monitoring	Data Logger (GL 220)
	Heater	Heating film (PTC)
	Temp. control	PID controller (NX_1)
	Power consumption	Power manager (B200)
	Pollutant	Loctite 401 (20 g)
	Monitoring	Three notebooks
	Sampler	Sampling tube
		Sterile syringe (100 mL)
		1005TLL 5.0 mL syringe

**Table 3:** Specifications of volatile organic compounds (VOC) analyzer (SGVA-P2).

Parameter	Description			
Measurement principle	Gas chromatography using semiconductor gas sensor			
Carrier gas	High purity cylinder air			
Sampling method	Manual sampling with a syringe (5 cc)			
Decomposition capability	0.1 ppb			
Measurement time	8 min			
Power supply	100–240 V, 50/60 Hz			
Measurement range	toluene	ethylbenzene	m-xylene	styrene
	5–1000 ppb	5–1000 ppb	5–1000 ppb	5–1000 ppb

**Table 4:** Boundary condition of CFD (computational fluid dynamics) analysis for bake-out.

Classification	ALT 1	ALT 2	ALT 3
Bake-out method	Natural ventilation 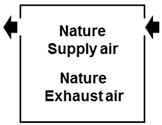	Mechanical ventilation 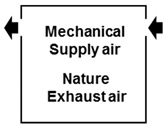	Installation of SBM 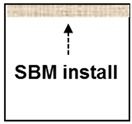
Inflow Boundary	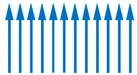 *U*_mean_ = 0.054 m/s, Air change rate = 0.5 h^−1^	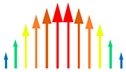 *U*_mean_ = 0.324 m/s, *U*_max_ = 0.53 m/s, Air change rate = 3.0 h^−1^	N/A
Outflow Boundary	*U*_out_ = outflow (Mass flow conservation)	*U*_out_ = outflow (Mass flow conservation)	
Installation of SBM	N/A	N/A	Ceiling (A = 3.24 m^2^)
Operating time	2 h	2 h	2 h
Energy consumption	N/A	Fan energy = 50 W	N/A
Turbulent model	Low Reynolds number k-ε turbulence model		
Number of meshes	Around 400,000		
Wall boundary	No-slip		
Wall temperature	The experimental result was set as the value of each wall surface temp. (Figure 4)		
Pollutant	Toluene (Molecular weight, 92.13 kg/kgmol)		
Time step size	30 s		

SBM: Sorptive building material.

**Table 5:** Comparison of VOCs concentrations for precision.

Compound	Retention Time (s)	Signal Strength (mV)	Measured Concentration (ppb)	RSD (%)
Toluene	92.16 ± 1.57 ^(1)^	154.42 ± 3.21	103.77 ± 2.96	2.07
Ethylbenzene	171.78 ± 1.44	132.03 ± 2.97	103.96 ± 3.01	2.25
m-Xylene	211.12 ± 0.95	135.80 ± 3.22	102.90 ± 3.39	2.37
Styrene	307.16 ± 1.78	72.92 ± 2.05	104.40 ± 3.99	2.82

^(1)^ Values are presented as mean ± standard deviation (*n* = 8).

**Table 6:** Comparison of VOCs concentrations for accuracy.

Compound	Injection Concentration (ppb)	Signal Strength (mV)	Measured Concentration (ppb)	Accuracy (%)	RSD (%)
Toluene	10	53.49 ± 0.46 ^(^^1)^	10.67 ± 0.42	102.11–112.10	0.86
	30	76.85 ± 0.82	32.22 ± 0.75	104.10–111.00	1.07
	100	158.95 ± 3.48	107.95 ± 3.21	104.80–111.20	2.19
	300	377.18 ± 3.66	309.25 ± 3.38	101.60–104.10	0.97
Ethylbenzene	10	39.55 ± 0.30	10.37 ± 0.30	102.00–108.00	0.77
	30	60.18 ± 0.79	31.25 ± 0.81	102.00–108.20	0.79
	100	133.10 ± 1.08	105.05 ± 1.09	103.60–105.90	0.10
	300	332.11 ± 2.84	306.45 ± 2.88	100.73–102.83	2.84
m-Xylene	10	47.82 ± 0.32	10.25 ± 0.34	98.00–105.90	0.67
	30	67.33 ± 0.34	30.80 ± 0.36	101.33–104.10	0.51
	100	139.70 ± 3.25	107.02 ± 3.42	102.40–110.00	2.32
	300	325.01 ± 1.65	302.15 ± 1.74	100.00–101.40	0.51
Styrene	10	24.70 ± 0.08	10.82 ± 0.17	108.00–110.00	0.35
	30	35.32 ± 0.43	31.42 ± 0.85	100.67–106.67	1.23
	100	74.96 ± 0.91	108.35 ± 1.78	106.80–110.90	1.22
	300	178.69 ± 1.46	309.65 ± 2.84	101.87–104.07	0.82

^(1)^ Values are presented as mean ± standard deviation (*n* = 4).

**Table 7:** Background concentration of test chamber.

Case	Set Temp. (°C)	Toluene (ppb)	Ethylbenzene (ppb)	m-Xylene (ppb)	o-Xylene (ppb)	Styrene (ppb)
Case 1	30	17.2	4.1	3.8	-	2.6
Case 2	40	18.4	3.7	5.6	-	4.3
Case 3	50	18.7	4.4	5.3	-	2.8

**Table 8:** Energy consumption and maximum concentration of toluene per case.

Case	*E_t_* (Rate of Increase)	*E_m_* (Rate of Increase)	Maximum Concentration of Toluene (Rate of Increase)
Case 1	974 Wh (-)	852 Wh (-)	41.0 ppb (-)
Case 2	3634 Wh (273%)	2755 Wh (223%)	59.4 ppb (45%)
Case 3	6789 Wh (597%)	5280 Wh (519%)	80.3 ppb (96%)

*E*_t_: Total electric energy consumption including the stabilization period. *E*_m_: Electric energy consumption during pollutant measurement period.

**Table 9:** Pollutant Concentration Reduction Effect per alternative (ALT).

ALT	*E_m_* with Fan Energy (Wh)	Age-of-Air (s)	Decreasing Rate of Concentration Per Energy Consumption (ppb/kWh)
ALT 1	5280	2156	10.68
ALT 2	5380	1632	11.34
ALT 3	5280	1936	11.30

*E*_m_: Electric energy consumption during pollutant measurement period.

## References

[1] Barabad M., Jung W., Versoza M., Kim M., Ko S., Park D., Lee K. (2018). Emission Characteristics of Particulate Matter, Volatile Organic Compounds, and Trace Elements from the Combustion of Coals in Mongolia. Int. Environ. Res. Public Health.

[2] Salthammer T., Mentese S., Marutzky R. (2010). Formaldehyde in the Indoor Environment. Chem. Rev..

[3] Smith M.T. (2010). Advances in Understanding Benzene Health Effects and Susceptibility. Annu. Rev. Public Health.

[4] Du Z., Mo J., Zhang Y. (2014). Risk assessment of population inhalation exposure to volatile organic compounds and carbonyls in urban China. Environ. Int..

[5] Girman J.R., Alevantis L.E., Kulasingam G.C., Petreas M.X., Webber L.M. (1989). The bake-out of an office building: A case study. Environ. Int..

[6] Girman J.R. (1989). Volatile organic compounds and building bake-out. Occup. Med..

[7] Lu Y., Liu J., Lu B.-N., Jiang A.-X., Zhang J.-R., Xie B., Li J., Zhang L. (2007). Numerical value research on bake-out technology with dilution ventilation for building materials. Proc. Build. Simul..

[8] Edwards R.D., Jurvelin J., Koistinen K., Saarela K., Jantunen M. (2001). VOC source identification from personal and residential indoor, outdoor and workplace microenvironment samples in EXPOLIS-Helsinki, Finland. Atmos. Environ..

[9] Kang D.H., Choi D.H., Lee S.M., Yeo M.S., Kim K.W. (2010). Effect of bake-out on reducing VOC emissions and concentrations in a residential housing unit with a radiant floor heating system. Build. Environ..

[10] Lv Y., Liu J., Wei S., Wang H. (2016). Experimental and simulation study on bake-out with dilution ventilation technology for building materials. J. Air Waste Manag. Assoc..

[11] Lee C., Haghighat F., Ghaly W. (2005). A study on VOC source and sink behavior in porous building materials-analytical model development and assessment. Indoor Air.

[12] Jiang C., Li D., Zhang P., Li J., Wang J., Yu J. (2017). Formaldehyde and volatile organic compound (VOC) emissions from particleboard: Identification of odorous compounds and effects of heat treatment. Build. Environ..

[13] Ataka Y., Kato S., Murakami S., Zhu Q., Ito K., Yokota T. (2004). Study of effect of adsorptive building material on formaldehyde concentrations; development of measuring methods and modeling of adsorption phenomena. Indoor Air.

[14] Seo J., Kato S., Ataka Y., Chino S. (2009). Performance test for evaluating the reduction of VOCs in rooms and evaluating the lifetime of sorptive building materials. Build. Environ..

[15] Park S., Seo J., Kim J.T. (2015). A study on the application of sorptive building materials to reduce the concentration and volume of contaminants inhaled by occupants in office areas. Energy Build..

[16] Graf M., Barrettino D., Taschini S., Hagleitner C., Hierlemann A., Baltes H. (2004). Metal oxide-based monolithic complementary metal oxide semiconductor gas sensor microsystem. Anal. Chem..

[17] Fine G.F., Cavanagh L.M., Afonja A., Binions R. (2010). Metal oxide semi-conductor gas sensors in environmental monitoring. Sensors.

[18] Kim S., Choi Y.-K., Park K.-W., Kim J.T. (2010). Test methods and reduction of organic pollutant compound emissions from wood-based building and furniture materials. Bioresour. Technol..

[19] Yang X. (1999). Study of Building Material Emissions and Indoor Air Quality. Ph.D. Thesis.

[20] ANSYS 17.2 (2011). Fluent Theory Guide.

[21] Hussain S., Oosthuizen P.H., Kalendar A. (2012). Evaluation of various turbulence models for the prediction of the airflow and temperature distributions in atria. Energy Build..

[22] Abe K., Kondoh T., Nagano Y. (1994). A new turbulence model for predicting fluid flow and heat transfer in separating and reattaching flows—I. Flow field calculations. Int. J. Heat Mass Transf..

[23] Bulińska A., Popiołek Z., Buliński Z. (2014). Experimentally validated CFD analysis on sampling region determination of average indoor carbon dioxide concentration in occupied space. Build. Environ..

[24] The Society of Chemical Engineers (1999). Handbook of Chemistry.

[25] Seo J., Lee S., Kim S., Nam Y. (2013). Control of emission rates of chemical compounds emitted by controlling their mass transfer coefficients on the surface of the tested building material. J. Adhes. Sci. Technol..

[26] Lim E.-S. (2013). Evaluation of Contaminant Concentration Reduction Effect of Sorptive Building Materials by New Ventilation Index-Net Escape Velocity. J. Archit. Inst. Korea Plan. Des..

[27] ICH Expert Working Group Validation of analytical procedures: Text and methodology Q2(R1). Proceedings of the International Conference on Harmonisation of Technical Requirements for Registration of Pharmaceuticals for Human Use.

[28] ASHRAE (2004). ASHRAE Standard 129—Measuring Air-Change Effectiveness.

[29] Piccolo A., Siclari R., Rando F., Cannistraro M. (2017). Comparative performance of thermoacoustic heat exchangers with different pore geometries in oscillatory flow. Implementation of experimental techniques. Appl. Sci..

